# Association between plasma vitamin B5 and coronary heart disease: Results from a case-control study

**DOI:** 10.3389/fcvm.2022.906232

**Published:** 2022-10-13

**Authors:** Pengfei Sun, Haoyu Weng, Fangfang Fan, Nan Zhang, Zhihao Liu, Ping Chen, Jia Jia, Bo Zheng, Tieci Yi, Yuxi Li, Yan Zhang, Jianping Li

**Affiliations:** ^1^Department of Cardiology, Peking University First Hospital, Beijing, China; ^2^School of Pharmacy, Jinan University, Guangzhou, China; ^3^Key Laboratory of Molecular Cardiovascular Science of Ministry of Education, Peking University, Beijing, China

**Keywords:** vitamin B5 (pantothenic acid), coronary heart disease, smoking, case control, L-shaped relationship

## Abstract

**Aim:**

The relationship of vitamin B5 and coronary heart disease (CHD) is still uncertain. This case–control study was performed to evaluate the relationship between the plasma vitamin B5 concentration and the risk of CHD.

**Materials and methods:**

The study involved 429 patients with >70% stenosis of the coronary arteries on coronary angiography and 429 matched controls were included for age ± 2 years, gender, and date of coronary angiography examination ± 180 days. Logistic regression analyses were performed to evaluate the association between plasma vitamin B5 and the risk of CHD.

**Results:**

An L-shaped relationship was found between the plasma vitamin B5 concentration and CHD. Compared with patients with low vitamin B5 (first quartile, <27.6 ng/ml), the odds ratio (OR) and 95% confidence interval (CI) for participants in the third quartile (34.9–44.0 ng/ml) and fourth quartile (≥44.0 ng/ml) were 0.42 (95% CI, 0.26–0.70) and 0.49 (95% CI, 0.29–0.82), respectively. In the threshold effect analysis, the risk of CHD significantly decreased as the vitamin B5 concentration increased (per 10 ng/ml increment: OR, 0.71; 95% CI, 0.57–0.89) in participants with a plasma vitamin B5 concentration of <40.95 ng/ml; however, an increased plasma vitamin B5 concentration was no longer associated with a decreased risk of CHD (per 10 ng/ml increment: OR, 1.00; 95% CI, 0.87–1.14) in participants with a plasma vitamin B5 concentration of ≥40.95 ng/ml. The association between vitamin B5 and CHD was stronger in ever or current smokers than non-smokers (*p*-interaction = 0.046).

**Conclusion:**

Plasma vitamin B5 has an L-shaped relationship with CHD, with a threshold around 40.95 ng/ml. This association was modified by smoking.

## Introduction

In spite of the improvements in prevention and treatment, coronary heart disease (CHD) remains one of the most common non-communicable diseases and leading cause of death globally (1). The burden of CHD is also increasing in Chinese population in recent decades (2). The prevalence of ischemic heart disease (IHD) nearly doubled since 1990, reaching approximately 23 million in 2016 (2).

At present, identification and management of traditional risk factors for CHD are the main focus of the clinical guidelines, including smoking, dyslipidemia, hypertension, obesity, diabetes, and physical inactivity (3–6). However, in light of rising CHD prevalence and recognition of residual risk despite the effort to control traditional risk factors, there is a clear need to explore non-traditional risk or protective factors. In this regard, a better understanding the role of micronutrients is highlighted by the US precision nutrition initiative (7).

Along this line, vitamin B5 is of considerable interest. It belongs to the family of water-soluble B vitamin, also known as pantothenic acid or pantothenate or “anti-stress vitamin” (8, 9). Vitamin B5 was first discovered by Williams et al. (10) in 1933 as an essential nutrient for yeast. One important fact is that vitamin B5 must be obtained from various dietary sources or intestinal bacteria, because human body cannot synthesize B5 (11). The vitamin B5 obtained from food is in the form of coenzyme A (12). Therefore, clinicians infrequently encounter vitamin B5 deficiency, the clinical manifestations of which include generalized malaise, burning foot syndrome (13), insomnia, and autoimmune arthritis (14). Vitamin B5 can also be obtained from intestinal bacterial sources, suggesting that the intestinal microbiome might impact the concentration of vitamin B5 in plasma (12, 15).

The importance of vitamin B5 is dictated by its role in cellular metabolism. It is the key precursor to the biosynthesis of coenzyme A, which is important in the synthesis and degradation of fatty acids, synthesis of phospholipids, synthesis of heme, and operation of the tricarboxylic acid cycle (16, 17). Furthermore, pantethine, the disulfide derivative of vitamin B5, can reduce the concentrations of low-density lipoprotein cholesterol (LDL-C), very-low-density lipoprotein (VLDL), total cholesterol, triglyceride, and apolipoprotein B and increase the concentrations of high-density lipoprotein cholesterol and apolipoprotein A-1 (18). Therefore, it has been regarded as a nutritional supplement in the United States since 1992 (15). Pantethine is well-tolerated and has a low occurrence (3.6%) of mild gastrointestinal adverse effects (19). Pantethine can also reportedly improve the function of platelet, exert an antioxidant effect, protect the endothelium (20, 21), modify lipid deposition, and reduce fatty streak formation in major arteries (18, 22). Vitamin B5 deficiency may be related to a relative “hyperadrenergic” state, increasing the risk of hypertension, arrhythmia, stroke, and some other diseases (14). Moreover, dexpanthenol, the precursor to vitamin B5, is reportedly beneficial in rats with ischemia–reperfusion injury of the brain and cardiovascular system (23). According to several studies, vitamin B5 might also be related to obesity (24) and visceral fat accumulation (VFA) (25). There are also reports on the association between B5 and hypertension (26, 27). Previous study had also stated a dietary multivitamin, multimineral and phytonutrient supplement, which contained vitamin B5, could lower homocysteine level (28).

When it comes to CHD, there are many unsettled questions regarding the role of vitamin B5. This case-control study sought to advance our understanding on the association of vitamin B5 with risk of CHD in Chinese men and women undergoing coronary angiography by involving patients with CHD and matched controls from hospital, with a particular attention to the effect modification by or joint effects with traditional CVD risk factors.

## Materials and methods

### Study participants

**FIGURE 1:**
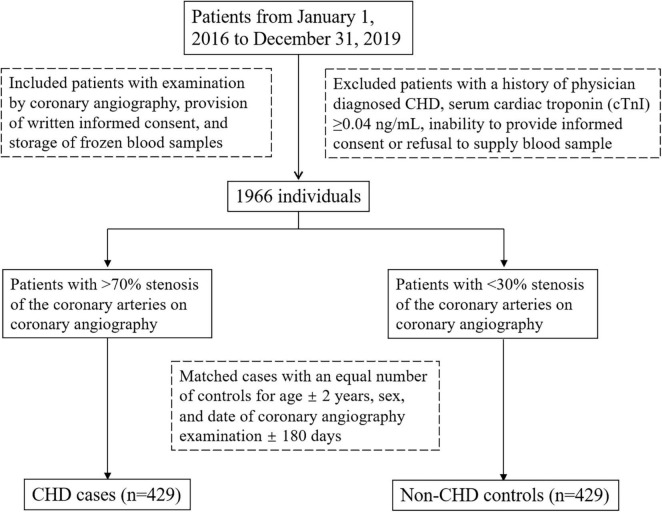
The flow chat of the enrollment.

Participants were enrolled from 1 January 2016 to 31 December 2019 in Peking University First Hospital, Beijing, China. A case-control study design was used. The inclusion criteria were examination by coronary angiography, provision of written informed consent, and storage of frozen blood samples in the Peking University First Hospital biorepository. Exclusion criteria were as follows: a history of physician diagnosed CHD; serum cardiac troponin (cTnI) ≥ 0.04 ng/ml; inability to provide informed consent; refusal to supply a venous blood sample. One thousand nine hundred and sixty six individuals were included. Then a CHD case was defined as a patient with >70% stenosis of the coronary arteries on coronary angiography and non-CHD controls were defined as a patient with <30% coronary stenosis. In total, we matched 429 patients with CHD with an equal number of controls for age ± 2 years, gender, and date of coronary angiography examination ±180 days. Figure 1 showed the flow chat of the enrollment. All participants provided written informed consent, and the study protocol was approved by the ethics committee of both Peking University and Peking University First Hospital. The study was conducted in accordance with the Declaration of Helsinki.

### Measurement of vitamin B5 and covariates

The plasma vitamin B5 concentration was measured using liquid chromatography tandem mass spectrometry. The linear range for plasma vitamin B5 was 2.5–1,000 ng/ml. The precision within and between batches was <15%.

For each participant, we reviewed the medical records and abstracted the following variables: gender; age; height; weight and body mass index (BMI); date of coronary angiography examination; systolic blood pressure (SBP); fasting plasma glucose (FPG) concentration; plasma creatinine concentration; plasma LDL-C concentration; plasma homocysteine concentration; smoking status (current, never, or ever); drinking status (current, never, or ever); diagnosis of hypertension, diabetes, and dyslipidemia; and use of antihypertensive, hypoglycemic, and lipid-lowering drugs.

### Statistical analysis

Continuous data are presented as mean ± standard deviation for normally distributed variables and as median (interquartile range) for non-normally distributed variables. Categorical variables are presented as number and percentage. Differences between two groups were compared using *t*-tests for normally distributed continuous variables including age, BMI, SBP, LDL-C, FPG, Crea; Mann–Whitney U-test for non-normally distributed continuous variables including homocysteine, VB5; and χ^2^ tests for categorical variables including smoking status, drinking status, diagnosis of disease, medication.

Odds ratios (ORs) and 95% confidence intervals (CIs) of CHD were estimated by modeling the plasma vitamin B5 concentration as continuous as well as categorical variables using conditional logistic regression, conditioned on the matching factors of age, gender, and operation time with and without adjusting for BMI; SBP; FPG; smoking status; drinking status; discharge diagnosis of hypertension, diabetes, and dyslipidemia; use of antihypertensive, hypoglycemic, and lipid-lowering drugs; LDL-C concentration, and plasma creatinine concentration. We also assessed the possible modifications of the association between the plasma vitamin B5 concentration and CHD by including interaction terms in the logistic regression models. Interactions between subgroups and the vitamin B5 concentration were examined by likelihood ratio testing. A smoothing function and two-piecewise linear regression model were used to examine the threshold effect of the plasma vitamin B5 concentration on CHD. A two-tailed *p*-value of <0.05 was considered statistically significant in all analyses, which were performed using R software version 3.6.3.

## Results

### Patient characteristics

This case–control study involved 429 cases and 429 matched controls and the characteristics of both groups were showed in Table 1. The median plasma vitamin B5 concentration was 33.3 ng/ml (interquartile range, 26.7–42.7 ng/ml) among CHD cases, and 36.9 ng/ml (interquartile range, 29.8–45.1 ng/ml) among controls. CHD cases had a higher FPG concentration, lower frequency of current smoking, lower plasma vitamin B5 concentration, and higher frequency of hypertension, diabetes, plasma homocysteine levels and hyperlipidemia. Participants’ characteristics at different plasma vitamin B5 concentrations are shown Supplementary Table 1, and participants’ characteristics stratified by smoking status are shown Supplementary Table 2.

**TABLE 1:** Characteristics of cases and controls.

Characteristics^a^	Total	Non-CHD controls	CHD cases	*P*-value^b^
*N*	858	429	429	
Female, *n* (%)	456 (53.1)	228 (53.1)	228 (53.1)	1
Age, years	63.5 ± 10.4	63.1 ± 10.3	63.9 ± 10.5	0.241
BMI, kg/m^2^	26.1 ± 3.7	26.2 ± 3.7	25.9 ± 3.7	0.298
SBP, mm Hg	133.0 ± 15.8	132.4 ± 15.6	133.6 ± 16.1	0.262
LDL-C, mmol/l	2.3 ± 0.8	2.4 ± 0.8	2.3 ± 0.8	0.05
FPG, mmol/l	7.0 ± 3.0	6.5 ± 2.5	7.5 ± 3.4	< 0.001
Crea, μmoI/l	82.0 ± 49.3	80.2 ± 53.3	83.8 ± 45.0	0.276
Homocysteine, μmoI/l	13.4 (10.3, 17.3)	13.5 (10.0, 17.7)	13.4 (10.5, 16.9)	0.859
**Smoking status, *n* (%)**				0.005
Never	499 (59.4)	272 (64.6)	227 (54.2)	
Ever	158 (18.8)	64 (15.2)	94 (22.4)	
Current	183 (21.8)	85 (20.2)	98 (23.4)	
**Drinking status, *n* (%)**				0.988
Never	590 (70.9)	297 (70.7)	293 (71.1)	
Ever	82 (9.9)	42 (10.0)	40 (9.7)	
Current	160 (19.2)	81 (19.3)	79 (19.2)	
**Diagnosis of disease, *n* (%)**				
Hypertension	600 (69.9)	280 (65.3)	320 (74.6)	0.003
Diabetes	361 (42.1)	139 (32.4)	222 (51.7)	< 0.001
Dyslipidemia	669 (78.0)	317 (73.9)	352 (82.1)	0.004
**Medication, *n* (%)**				
Antihypertensive	476 (55.5)	226 (52.7)	250 (58.3)	0.099
Hypoglycemia	263 (30.7)	97 (22.6)	166 (38.7)	< 0.001
Lipid-lowering	411 (47.9)	171 (39.9)	240 (55.9)	< 0.001
VB5, ng/ml	34.9 (27.6, 44.0)	36.9 (29.2, 44.7)	33.3 (26.7, 42.7)	0.006

^a^Normally distributed variables were presented as mean ± standard deviation, non-normally were presented as median (interquartile range) and categorical variables are presented as number and percentage. ^b^Differences between two groups were compared using *t*-tests for normally distributed continuous variables, Mann–Whitney U-test for non-normally distributed continuous variables and χ^2^ tests for categorical variables. VB5, vitamin B5; CHD, coronary heart disease; BMI, body mass index; SBP, systolic blood pressure; LDL-C, low-density lipoprotein cholesterol; FPG, fast plasma glucose; Crea, plasma creatinine; HTD, hypertension drugs.

### Association between plasma vitamin B5 and coronary heart disease

**FIGURE 2:**
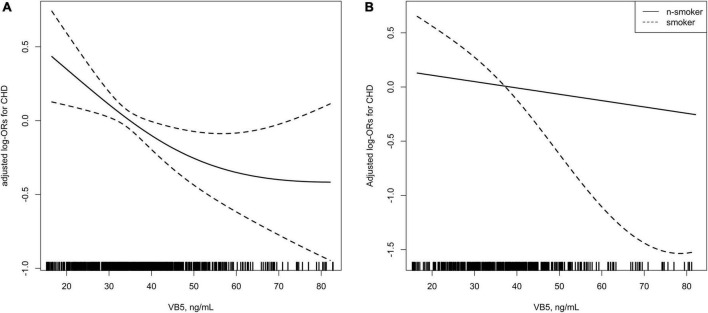
**(A)** Smoothing curve showing the effects of plasma vitamin B5 on the risk of coronary heart disease (CHD). **(B)** Smoothing curve showing the effects of plasma vitamin B5 on the risk of CHD stratified by smoking status.

In Figure 2 we showed the smoothing curve between the plasma vitamin B5 concentration and CHD in overall sample (Figure 2A) and stratified by smoking status (Figure 2B), adjusted for gender; age; BMI; SBP; FPG concentration; drinking status; discharge diagnosis of hypertension, diabetes, and dyslipidemia; LDL-C concentration; and plasma creatinine concentration. The smooth curve shows an L-shaped relationship between the plasma vitamin B5 concentration and CHD in overall sample.

In Table 2 we summarized the univariate and multivariate OR (95% CI) of CHD in relation to the plasma vitamin B5 concentration (as a categorical variable and in quartiles) in overall sample and stratified by gender. In overall sample, compared with patients with a low vitamin B5 concentration (first quartile, <27.6 ng/ml), the OR (95% CI) for participants in the third quartile (34.9–44.0 ng/ml) and fourth quartile (≥44.0 ng/ml) was 0.42 (95% CI, 0.26–0.70) and 0.49 (95% CI, 0.29–0.82), respectively. In males, compared with patients with a low vitamin B5 concentration (first quartile, <27.6 ng/ml), the OR (95% CI) for participants in the third quartile (34.9–44.0 ng/ml) and fourth quartile (≥44.0 ng/ml) was 0.27 (95% CI, 0.12–0.62) and 0.29 (95% CI, 0.13–0.66), respectively. In females, this association was insignificant, although the gender interaction effect was insignificant (*p* = 0.067).

**TABLE 2:** Association of vitamin B5 with risk of coronary heart disease (CHD).

VB5, ng/ml	Cases (%)	Model 1^a^		Model 2^b^	
		OR (95% CI)	*P*-value	OR (95% CI)	*P*-value
**Overall sample**					
**Quartiles**					
Q1 (<27.6 ng/ml)	126 (58.6)	Ref		Ref	
Q2 (27.6–34.9 ng/ml)	110 (51.4)	0.77 (0.53, 1.11)	0.161	0.70 (0.42, 1.15)	0.155
Q3 (34.9–44.0 ng/ml)	94 (43.9)	0.55 (0.38, 0.81)	0.003	0.42 (0.26, 0.70)	< 0.001
Q4 (=44.0 ng/ml)	99 (46.0)	0.61 (0.41, 0.89)	0.010	0.49 (0.29, 0.82)	0.007
**Categories**					
Q1 (<27.6 ng/ml)	126 (58.6)	Ref		Ref	
Q2–Q4 (≥27.6 ng/ml)	303 (47.1)	0.64 (0.47, 0.88)	0.005	0.53 (0.35, 0.80)	0.002
**Male**					
**Quartiles**					
Q1 (<27.6 ng/ml)	71 (65.1)	Ref		Ref	
Q2 (27.6–34.9 ng/ml)	50 (50.0)	0.54 (0.31, 0.95)	0.034	0.40 (0.19, 0.87)	0.020
Q3 (34.9–44.0 ng/ml)	39 (41.9)	0.36 (0.20, 0.66)	< 0.001	0.27 (0.12, 0.62)	0.002
Q4 (≥44.0 ng/ml)	41 (41.0)	0.36 (0.20, 0.64)	< 0.001	0.29 (0.13, 0.66)	0.003
**Categories**					
Q1 (<27.6 ng/ml)	71 (65.1)	Ref		Ref	
Q2–Q4 (≥27.6 ng/ml)	130 (44.4)	0.42 (0.26,0.68)	< 0.001	0.32 (0.17,0.62)	< 0.001
**Female**					
**Quartiles**					
Q1 (<27.6 ng/ml)	55 (51.9)	Ref		Ref	
Q2 (27.6–34.9 ng/ml)	60 (52.6)	1.04 (0.64, 1.72)	0.865	1.17 (0.56, 2.44)	0.669
Q3 (34.9–44.0 ng/ml)	55 (45.5)	0.77 (0.46, 1.28)	0.317	0.55 (0.27, 1.12)	0.102
Q4 (≥44.0 ng/ml)	58 (50.4)	0.95 (0.57, 1.60)	0.852	0.83 (0.38, 1.80)	0.643
**Categories**					
Q1 (<27.6 ng/ml)	55 (51.9)	Ref		Ref	
Q2–Q4 (≥27.6 ng/ml)	173 (49.4)	0.91 (0.60, 1.38)	0.673	0.79 (0.44, 1.41)	0.419

^a^Conditioned on the matching factors of age, gender, and operation time. ^b^Conditioned on the matching factors of age, gender, and operation time and adjusted for BMI; SBP; fasting plasma glucose concentration; smoking status; drinking status; diagnosis of hypertension, diabetes, and dyslipidemia; LDL-C concentration; plasma creatinine concentration; and use of antihypertensive, hypoglycemic and lipid-lowering medications. VB5, vitamin B5; CHD, coronary heart disease; OR, odds ratio; CI, confidence interval; Ref, reference; BMI, body mass index; SBP, systolic blood pressure; LDL-C, low-density lipoprotein cholesterol.

Although higher plasma vitamin B5 quartiles were associated with a lower frequency of CHD, a threshold effect was found for this association. In the threshold effect analysis, the risk of CHD significantly decreased as the plasma vitamin B5 concentration increased (per-10 ng/ml increment: OR, 0.71; 95% CI, 0.57–0.89) in participants with a plasma vitamin B5 concentration of <40.95 ng/ml; however, increased plasma vitamin B5 was no longer associated with a decreased risk of CHD (per 10 ng/ml increment: OR, 1.00; 95% CI, 0.87–1.14) in participants with a plasma vitamin B5 concentration of ≥40.95 ng/ml. The *p* of the log likelihood ratio test comparing the two-piecewise model with the linear logistic regression model was 0.026 (Table 3).

**TABLE 3:** Threshold effect analysis of vitamin B5 (per 10 ng/ml) on coronary heart disease (CHD) using piecewise logistic regression.

Threshold effect analysis	B	SE	Exp (B)	*Z* value	OR (95% CI)	*P*
**Model comparison**						
**Model I**						
One line model	−0.12	0.05	0.89	−2.22	0.89 (0.80, 0.99)	0.026
**Model II**						
VB5 turn point (K)					40.95 ng/ml	
<K, effect 1	−0.34	0.11	0.71	−2.98	0.71 (0.57, 0.89)	0.003
>K, effect 2	0	0.07	1	−0.07	1.00 (0.87, 1.14)	0.945
**Log likelihood ratio test *P*-value**					0.026	

Adjustment was performed for gender; age; BMI; SBP; fasting plasma glucose concentration; drinking status; diagnosis of hypertension, diabetes, and dyslipidemia; LDL-C concentration; plasma creatinine concentration; and use of antihypertensive, hypoglycemic, and lipid-lowering medications. CHD, coronary heart disease; OR, odds ratio; CI, confidence interval; VB5, vitamin B5; BMI, body mass index; SBP, systolic blood pressure; LDL-C, low-density lipoprotein cholesterol. A smoothing function and two-piecewise logistics regression model were used to examine the threshold effect of the plasma VB5 concentration on CHD. The turning point was determined using trial and error, including selection of turning points along a predefined interval and then choosing the turning point that gave the maximum model likelihood. A log likelihood ratio test was used to compare the two-piecewise model with the linear logistic regression model.

### Subgroup and interaction analyses

In the stratified analyses to assess the relationship between the plasma vitamin B5 concentration (<27.6 vs. ≥27.6 ng/ml) and CHD in the various subgroups, none of the following variables significantly modified the association between vitamin B5 and CHD: age (<60 vs. ≥60 years, *p*-interaction = 0.346); gender (*p*-interaction = 0.067); BMI (<24, 24–28, and ≥28 kg/m^2^, *p*-interaction = 0.698); alcohol drinking status (never, ever, or current; *p*-interaction = 0.402); plasma creatinine concentration (<82 vs. ≥82 μmol/l, *p*-interaction = 0.238); and diagnosis of hypertension (no or yes, *p*-interaction = 0.837), diabetes (no or yes, *p*-interaction = 0.686), and dyslipidemia (no or yes, *p*-interaction = 0.807). However, the smoking status modified the association between vitamin B5 and the risk of CHD, the association between vitamin B5 and CHD was stronger in ever or current smokers than non-smokers (*p*-interaction = 0.046, Table 4). Participants’ characteristics stratified by smoking status are shown Supplementary Table 2.

**TABLE 4:** Subgroup and interaction analyses for the association between vitamin B5 (quartiles 2–4 vs. 1) and coronary heart disease (CHD).

Subgroup	B5 Q1 (<27.6 ng/ml) case (%)	B5 Q2–4 (≥27.6 ng/ml) case (%)	OR (95% CI)	*P*	*P*-interaction
**Gender**					0.067
Male	71 (65.1)	130 (44.4)	0.39 (0.23, 0.67)	< 0.001	
Female	55 (51.9)	173 (49.4)	0.77 (0.47, 1.26)	0.302	
**Age, years**					0.346
<60	55 (59.1)	76 (41.5)	0.45 (0.25, 0.80)	0.007	
≥60	71 (58.2)	227 (49.3)	0.64 (0.40, 1.01)	0.054	
**BMI, Kg/m^2^**					0.698
<24	38 (58.5)	86 (51.8)	0.69 (0.35, 1.34)	0.271	
24–28	58 (59.2)	134 (46.9)	0.48 (0.28, 0.82)	0.007	
≥28	27 (56.2)	80 (44.2)	0.59 (0.29, 1.22)	0.155	
**Crea, μ mol/l**					0.238
<82	89 (57.4)	174 (43.7)	0.48 (0.31, 0.74)	< 0.001	
≥82	37 (62.7)	128 (52.9)	0.78 (0.40, 1.53)	0.468	
**Smoking status**					0.046
Never	50 (45.5)	177 (45.5)	0.82 (0.51, 1.31)	0.402	
Ever	29 (74.4)	65 (54.6)	0.26 (0.10, 0.67)	0.006	
Current	42 (70)	56 (45.5)	0.39 (0.19, 0.81)	0.011	
**Drinking status**					0.402
Never	80 (54.1)	213 (48.2)	0.65 (0.42, 0.99)	0.046	
Ever	14 (70)	26 (41.9)	0.30 (0.09, 1.02)	0.054	
Current	26 (65)	53 (44.2)	0.44 (0.19, 1.01)	0.052	
**Hypertension**					0.837
No	43 (53.1)	66 (37.3)	0.53(0.29, 0.97)	0.04	
Yes	83 (61.9)	237 (50.9)	0.58(0.37, 0.91)	0.019	
**Diabetes**					0.686
No	70 (49.3)	137 (38.6)	0.59 (0.38, 0.92)	0.02	
Yes	56 (76.7)	166 (57.6)	0.51 (0.27, 0.96)	0.036	
**Dyslipidemia**					0.807
No	25 (51)	52 (37.1)	0.52 (0.25, 1.09)	0.084	
Yes	101 (60.8)	251 (49.9)	0.58 (0.38, 0.87)	0.009	

Adjusted, if not stratified, for gender; age; BMI; SBP; fasting plasma glucose concentration; smoking status; drinking status; diagnosis of hypertension, diabetes, and dyslipidemia; LDL-C concentration; plasma creatinine concentration; and use of antihypertensive, hypoglycemic, and lipid-lowering medications. OR, odds ratio; CI, confidence interval; CHD, coronary heart disease; BMI, body mass index; SBP, systolic blood pressure; LDL-C, low-density lipoprotein cholesterol; FPG, fasting plasma glucose; Crea, plasma creatinine; Q1, quartile 1; Q2–4, quartile 2–4.

## Discussion

To our knowledge, there are limited studies that specifically examined vitamin B5 and CHD. From mechanistic perspective, our study findings are biologically plausible. The possible mechanism between vitamin B5 and CHD can be postulated to involve inflammatory events. It is widely accepted that CHD manifests with chronic low-grade inflammation, while vitamin B5 has an antioxidant effect in the inflammatory process underlying the pathogenesis of atherosclerosis (8). Jung et al. (29) reported that high dietary vitamin B5 intake was significantly related to a lower serum C-reactive protein (CRP) concentration at the 5 years follow-up in South Korea; CRP can directly bind LDL-C and is involved in the formation of foam cells in the inflammatory process. Additionally, the findings of Scheurig et al. (30) indicated that multivitamin supplements, including vitamin B5, were associated with lower CRP concentrations among female patients. He et al. (31) stated that vitamin B5 might promote neutrophils to secrete anti-inflammatory cytokines to reduce the recruitment or activation of macrophages in early infection. In this way, vitamin B5 might regulate the macrophages in the early process of atherosclerosis.

In support of our findings, previous studies showed that vitamin B5 is also associated with several risk factors of CHD, such as dyslipidemia, hypertension, and obesity. An association between vitamin B5 and the lipid-regulating effect of pantethine has been supported by some experiments (18, 19). A randomized controlled trial by Evans et al. (15) also suggested that pantethine, synthesized from vitamin B5 in the body, can lower the concentrations of total cholesterol, LDL-C, very-low-density lipoprotein, and triglycerides in patients with a low to moderate risk of CVD. Although the mechanism underlying the ability of pantethine to reduce blood lipids is unclear, dyslipidemia plays a major role in the development of CVD (32). In the present study, the diagnosis of CHD in all participants was based on coronary angiography, which is more accurate than other methods.

Vitamin B5 was also proven to be associated with hypertension in some animal experiments (33–35). Koyanagi et al. (26) reported that residents with lower serum vitamin B5 concentrations living in rice field areas were more likely to be diagnosed with hypertension than residents with higher serum vitamin B5 concentrations living in upland villages in Japan, this relationship was also confirmed by a survey launched in black South African children (27).

There is also some evidence of a relationship between vitamin B5 and obesity. According to a survey among male adolescents (24), higher dietary vitamin B5 intake was associated with a greater likelihood of obesity prevention in male adolescents. Another study in Japan (25) focused on the relationship between vitamin B5 and VFA, a persuasive predictor of CVD. One of the conclusions was that vitamin B5 was significantly inversely correlated with VFA. These associations between vitamin B5 and CHD risk factors may also help explain the association between vitamin B5 and CHD.

The correlation between B vitamin supplementation and its protective effect against CVD seems to be attributed to a decreased serum homocysteine (Hcy) concentration (28), which is harmful to the myocardium at a high level (36). However, no previous studies have directly investigated the relationship between vitamin B5 and Hcy or CHD. The mechanisms underlying this correlation are not well-established. The above-mentioned study was based on multivitamin supplements which contained vitamin B5. Therefore, the effect of reducing homocysteine may be the joint effect of multiple vitamins supplementation. We further examined the relationship between vitamin B5 and homocysteine, and the results showed that the two were not related. More qualified researches are needed to investigate the relationship of vitamin B5 and homocysteine.

We also observed a stronger correlation in smokers than non-smokers (*p* for interaction = 0.046). Smoking was related to the increasement of activation of neutrophil and macrophages (37). And vitamin B5 could reduce the recruitment or activation of macrophages (31), which could be an explanation of the interaction of smoking status and vitamin B5.

In order to make the characteristics between CHD cases and controls more differentiated, we included patients with severe coronary artery stenosis (>70% stenosis of the coronary arteries on coronary angiography) as CHD cases. In this way, we hoped to make the relationship between vitamin B5 and coronary heart disease more apparent and easier to discover. The clinical situations of these patients with elevated myocardial enzymes were more complex. These patients may have acute coronary syndrome, cardiomyopathy, or heart failure and the vitamin status could be affected (38, 39). So, we excluded these patients.

Our study findings have several strengths. Vitamin B5 was previously hardly considered to be associated with CHD and our study implied the potential correlations. This could provide us a new point of view to understand the mechanism of CHD. Our study was well-designed by including a relatively large number of patients with comprehensive clinical data, which made the results more credible.

A few limitations of this study require mentioning. First, this is a case–control study, and we can’t get a causal relationship between vitamin B5 and CHD. Hence, further epidemiological studies and subsequent clinical trials should be conducted to examine the role of vitamin B5 in the development of CHD. Second, selection bias inevitably exists, although we used strict statistical matching of age, gender, and the date of the coronary angiography examination to minimize confounders and maximize comparability. Third, methodological biases exist. We could not include all the confounders in the regression models. But we looked through all the variables that we could get from the electrical medical records and include variables that could have an effect on vitamin B5 or CHD. Fourth, our study is limited by the lack of long-term observation of the plasma vitamin B5 concentration. Vitamin B5 was measured only once, and potential fluctuation was not taken into account. Fifth, we did not check the vitamin B5 intake in everyday diet, and this might cause the bias. Finally, the study was carried out in a hospital setting containing only Chinese men and women, the generalizability of the study should be assessed when it applies to other populations with different characteristics in future work.

Our findings, if further confirmed by future studies, offers a new venue for preventing or treating CHD, given that vitamin B5 supplementation is simple, safe, and inexpensive. According to NIH website (40), people can obtain pantothenic acid by eating a variety of foods, including beef, poultry, seafood, organ meats, eggs and milk, vegetables such as mushrooms (especially shiitakes), avocados, potatoes, and broccoli, whole grains, such as whole wheat, brown rice and oats, peanuts, sunflower seeds, and chickpeas. If the “L” shaped association is true, it also implies that finding a dose that is appropriate for a patient is needed.

## Conclusion

In summary, this hospital-based case-control study showed a significant L-shaped relationship of the plasma vitamin B5 concentration with CHD, with a threshold around 40.95 ng/ml. The smoking status significantly modified this association, which was stronger in ever or currently smoking participants. Our findings provided a new idea for preventing or treating CHD and further studies are needed to confirm.

## Data availability statement

The raw data supporting the conclusions of this article will be made available by the authors, without undue reservation.

## Ethics statement

The studies involving human participants were reviewed and approved by Ethics review board of Peking University First Hospital. The patients/participants provided their written informed consent to participate in this study.

## Author contributions

JL and YZ designed the research and had primary responsibility for final content. ZL, JJ, BZ, and TY conducted the research. NZ provided the essential reagents. PS, FF, and YL analyzed the data and performed the statistical analysis. PS and HW wrote the manuscript. PC was responsible for the test of vitamin B5. All authors contributed to the article and approved the submitted version.
