# Supplementary material for: Association between plasma vitamin B5 and coronary heart disease: Results from a case-control study
Source: Front Cardiovasc Med. 2022 Oct 13;9:906232. doi: 10.3389/fcvm.2022.906232 (PMC9606243; doi:10.3389/fcvm.2022.906232)
Supplement: Supplementary file 1 [file Data_Sheet_1.docx]

**Supplemental Table 1. Characteristics of participants stratified by vitamin B5 quartiles**

| Characteristics | Q1 | Q2 | Q3 | Q4 | *p* value^a^ |
| --- | --- | --- | --- | --- | --- |
| N | 215 | 214 | 214 | 215 |  |
| Female, n (%) | 106(49.3) | 114(53.3) | 121(56.5) | 115(53.5) | 0.517 |
| Age, years | 60.5±10.3 | 62.8±10.1 | 65.3±9.7 | 65.3±10.7 | <0.001 |
| BMI, kg/m^2^ | 25.7±3.6 | 26.0±3.6 | 26.3±3.8 | 26.2±3.7 | 0.385 |
| SBP, mm Hg | 131.8±15.1 | 131.7±15.7 | 132.3±15.0 | 136.2±17.1 | 0.009 |
| LDL-C, mmol/L | 2.4±0.8 | 2.4±0.8 | 2.3±0.8 | 2.3±0.8 | 0.095 |
| FPG, mmol/L | 6.7±2.6 | 7.0±3.0 | 7.2±3.3 | 7.0±3.2 | 0.328 |
| Crea, μmoI/L | 73.3±17.4 | 78.2±18.1 | 77.1±19.1 | 99.6±91.6 | <0.001 |
| Smoking, n (%) |  |  |  |  | 0.067 |
| Never | 110(52.6) | 119(58.3) | 142(66.4) | 128(60.1) |  |
| Ever | 39(18.7) | 40(19.6) | 38(17.8) | 41(19.2) |  |
| Current | 60(28.7) | 45(22.1) | 34(15.9) | 44(20.7) |  |
| Drinking, n (%) |  |  |  |  | 0.743 |
| Never | 148(71.2) | 150(73.5) | 153(72.5) | 139(66.5) |  |
| Ever | 20(9.6) | 18(8.8) | 18(8.5) | 26(12.4) |  |
| Current | 40(19.2) | 36(17.6) | 40(19.0) | 44(21.1) |  |
| Prevalence of disease, n (%) | | | | | |
| Hypertension | 134(62.3) | 139(65.0) | 152(71.0) | 175(81.4) | <0.001 |
| Diabetes | 73(34.0) | 88(41.1) | 98(45.8) | 102(47.4) | 0.022 |
| Dyslipidemia | 166(77.2) | 160(74.8) | 171(79.9) | 172(80.0) | 0.506 |
| Medication, n (%) |  |  |  |  |  |
| Antihypertensive | 98(45.6) | 110(51.4) | 120(56.1) | 148(68.8) | <0.001 |
| Hypoglycemia | 49(22.8) | 65(30.4) | 70(32.7) | 79(36.7) | 0.015 |
| Lipid-lowering | 99(46.0) | 89(41.6) | 111(51.9) | 112(52.1) | 0.087 |
| VB5, ng/mL | 23.6  [20.2,25.8] | 31.1  [29.9,33.0] | 39.0  [37.2,41.0] | 52.5  [47.5,62.9] | <0.001 |

^a^ Differences between three groups were compared using ANOVA for normally distributed continuous variables, Mann–Whitney U-test for non-normally distributed continuous variables and χ2 tests for categorical variables.

Abbreviations: CHD, coronary heart disease; BMI, body mass index; SBP, systolic blood pressure; LDL-C, low density lipoprotein cholesterol; FPG, fast plasma glucose; Crea, plasma creatinine.

For continuous variables, data were presented as mean (SD) or median [inter-quartile range] depending on the distribution.

**Supplemental Table 2. Characteristics of participants stratified by current smoking status**

| Characteristics | Never | Ever | Current | *p* value^a^ |
| --- | --- | --- | --- | --- |
| N | 499 | 158 | 183 |  |
| Female | 403(80.8) | 14(8.9) | 28(15.3) | <0.001 |
| Age, years | 65.5±9.6 | 63.1±10.5 | 58.0±10.5 | <0.001 |
| BMI, kg/m^2^ | 26.0±3.9 | 26.5±3.3 | 25.9±3.5 | 0.271 |
| SBP, mm Hg | 133.6±15.8 | 132.8±16.9 | 131.7±14.8 | 0.365 |
| LDL-C, mmol/L | 2.4±0.8 | 2.2±0.8 | 2.4±0.9 | 0.185 |
| FPG, mmol/L | 7.1±3.3 | 7.0±2.9 | 6.5±2.2 | 0.093 |
| Crea, μmoI/L | 75.9±42.7 | 97.8±76.9 | 85.5±31.6 | <0.001 |
| Drinking status, n (%) | |  |  | <0.001 |
| Never | 452(91.5) | 57(37.0) | 78(43.1) |  |
| Ever | 12(2.4) | 49(31.8) | 21(11.6) |  |
| Current | 30(6.1) | 48(31.2) | 82(45.3) |  |
| Prevalence of disease, n (%) | | |  |  |
| Hypertension | 359(71.9) | 110(69.6) | 115(62.8) | 0.073 |
| Diabetes | 215(43.1) | 65(41.1) | 71(38.8) | 0.593 |
| Dyslipidemia | 390(78.2) | 127(80.4) | 139(76.0) | 0.615 |
| Medication, n (%) |  |  |  |  |
| Antihypertensive | 290(58.1) | 89(56.3) | 89(48.6) | 0.086 |
| Hypoglycemia | 165(33.1) | 46(29.1) | 51(27.9) | 0.354 |
| Lipid-lowering | 248(49.7) | 78(49.4) | 81(44.3) | 0.438 |
| VB5, ng/mL | 36.2[28.6,44.3] | 34.9[27.7,45.2] | 32.4[26.1,42.7] | 0.037 |

^a^ Differences between three groups were compared using ANOVA for normally distributed continuous variables, Mann–Whitney U-test for non-normally distributed continuous variables and χ2 tests for categorical variables.

Abbreviations: CHD, coronary heart disease; BMI, body mass index; SBP, systolic blood pressure; LDL-C, low density lipoprotein cholesterol; FPG, fast plasma glucose; Crea, plasma creatinine.

For continuous variables, data were presented as mean ± SD or median [inter-quartile range] depending on the distribution.
